# Rejecting Novel Motions in High-Density Myoelectric Pattern Recognition Using Hybrid Neural Networks

**DOI:** 10.3389/fnbot.2022.862193

**Published:** 2022-03-28

**Authors:** Le Wu, Xun Chen, Xiang Chen, Xu Zhang

**Affiliations:** School of Information Science and Technology, University of Science and Technology of China, Hefei, China

**Keywords:** human-machine interactions, novelty detection, neural networks, electromyography, pattern recognition

## Abstract

The objective of this study is to develop a method for alleviating a novel pattern interference toward achieving a robust myoelectric pattern-recognition control system. To this end, a framework was presented for surface electromyogram (sEMG) pattern classification and novelty detection using hybrid neural networks, i.e., a convolutional neural network (CNN) and autoencoder networks. In the framework, the CNN was first used to extract spatio-temporal information conveyed in the sEMG data recorded *via* high-density (HD) 2-dimensional electrode arrays. Given the target motion patterns well-characterized by the CNN, autoencoder networks were applied to learn variable correlation in the spatio-temporal information, where samples from any novel pattern appeared to be significantly different from those from target patterns. Therefore, it was straightforward to discriminate and then reject the novel motion interferences identified as untargeted and unlearned patterns. The performance of the proposed method was evaluated with HD-sEMG data recorded by two 8 × 6 electrode arrays placed over the forearm extensors and flexors of 9 subjects performing seven target motion tasks and six novel motion tasks. The proposed method achieved high accuracies over 95% for identifying and rejecting novel motion tasks, and it outperformed conventional methods with statistical significance (*p* < 0.05). The proposed method is demonstrated to be a promising solution for rejecting novel motion interferences, which are ubiquitous in myoelectric control. This study will enhance the robustness of the myoelectric control system against novelty interference.

## Introduction

Surface electromyograms (sEMG) have become a popular choice for controlling powered prosthetic devices because it can directly reflect the neural and muscular activities associated with motion intentions in a non-intrusive way (Parker et al., 2006). In the last few decades, a considerable number of studies have focused on the sEMG-based man–machine interface. Hand prosthesis (Hahne et al., 2018; Li et al., 2021), exoskeleton robots (Shi et al., 2021), and wearable devices (Moin et al., 2021) frequently use sEMG as a control source. Traditionally, myoelectric control was implemented using a pair of antagonistic muscles, each of which corresponded to one degree of freedom (DoF) based on their sEMG amplitudes (Finley and Wirta, 1967; Childress, 1969). However, few DoFs can be controlled due to the requirement of various muscle pairs. For decades, myoelectric pattern recognition (MPR) has garnered substantial attention since it enables dexterous control of multiple DoFs. The MPR technique utilizes machine learning algorithms to classify motion tasks based on statistical representations from sEMG signals. Many studies have asserted high classification accuracies for recognizing multiple motion tasks with several techniques or their combinations, including pre-processing, feature engineering, classification, and post-processing (Hudgins et al., 1993; Engelhart et al., 1999; Englehart and Hudgins, 2003; Hargrove et al., 2010; Zhang and Zhou, 2012; Chen and Wang, 2013; Wang et al., 2016; Zhang et al., 2017). Although promising outcomes have been reported in well-controlled laboratory settings, the MPR approach has yet to be widely used in clinical settings. As many studies stated, some issues including novel motion interference (Scheme et al., 2011; Tomczyński et al., 2015; Ding et al., 2019), electrode shift (Hargrove et al., 2008; He and Zhu, 2017), or variation in force and limb orientation (Al-Timemy et al., 2015; Cheng et al., 2018; Nougarou et al., 2019) can significantly decay practical performance of the MPR systems. Among them, the novel motion interference is a critical issue for implementing a MPR system in real-world scenarios.

**Figure 1:**
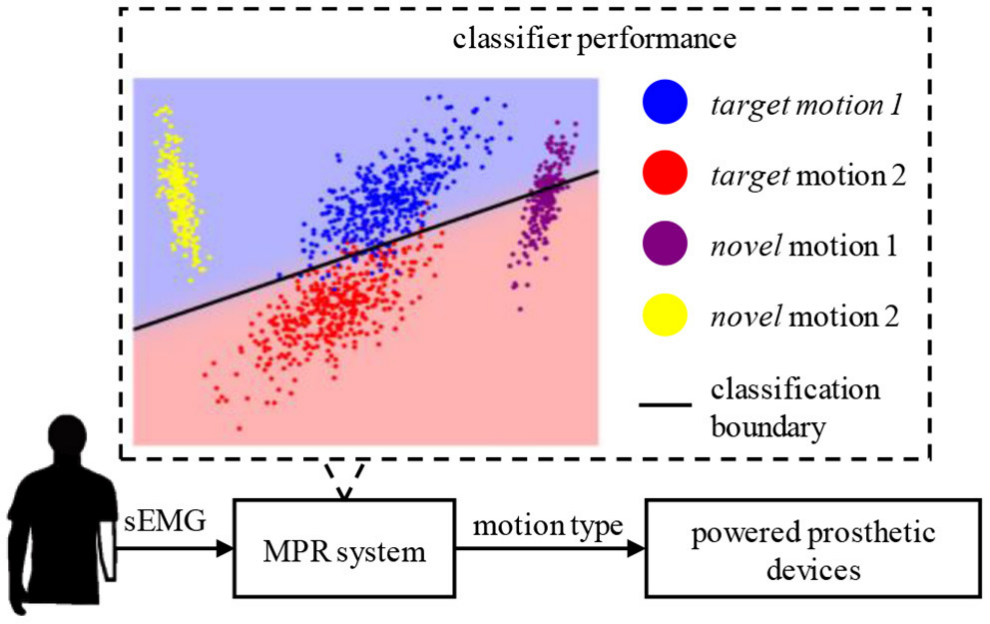
A scenario of novel motion interference involved in routine myoelectric pattern recognition (MPR) system.

In most typical MPR systems, classifiers are always trained with a restricted set of patterns and can then classify data samples from those patterns. However, a large number of motion patterns, instead of a specific few, may be involved by users in their daily life activities. As a result, the trained classifier in the MPR system may unavoidably fail to identify any unlearned pattern. These unlearned patterns, namely novel motion tasks, may probably be classified as one of the learned patterns by mistake. Figure 1 illustrates that, although the classifier can map the samples from two target motion tasks (blue and red dots) to two categories well, the samples from novel motion tasks (purple and yellow dots) that are not fed to the classifier in the training procedure fall into one part of the boundary erroneously. Accidental incorrect predictions of MPR systems that predominantly deliver negative feedback are undoubtedly a cause of frustration and discouragement. Therefore, it is critical to reject novel motion tasks in MPR systems.

Many studies in this area have been conducted to alleviate the novel motion interference (Liu and Huang, 2009; Scheme et al., 2011, 2013; Amsuess et al., 2015; Tomczyński et al., 2015; Ding et al., 2017, 2019; Robertson et al., 2019). Generally speaking, common solutions to this issue can be explained to design a filtering scheme that could only pass through target motion patterns while rejecting any unlearned novel motion patterns. The implementation concepts of these filtering schemes can be divided into two categories: one is based on confidence (Scheme et al., 2013; Tomczyński et al., 2015; Robertson et al., 2019) and the other uses a one-vs.-all classification rule (Liu and Huang, 2009; Scheme et al., 2011; Amsuess et al., 2015; Ding et al., 2017, 2019). As representative examples of the confidence-based schemes, Scheme et al. (2013) and Robertson et al. (2019) utilized the classifier to provide a confidence score for each decision and subsequently rejected samples from novel motion tasks with a score below the threshold. Similarly, Tomczyński et al. (2015) used the output entropy function of an artificial neural network to discriminate between target and novel motion tasks. The basic idea of the one-vs.-all classification rule is to model each known class, i.e., target motion task, by primarily learning from a training set containing only the samples of that class, and then a test sample will be rejected if it does not belong to any known class. Liu and Huang (2009) and Ding et al. (2019) suggested using an algorithm termed support vector data description (SVDD) to build multiple one-vs.-all classifiers. Scheme et al. (2011) employed a multiclass binary classification combined with a majority voting method to reject samples of novel motion tasks. Recently, Amsuess et al. (2015) and Ding et al. (2017) suggested an efficient rejection architecture based on the Fisher linear discriminant analysis (LDA) and the Mahalanobis distance (MD), where the MD was used to cast-off interference of novel motion tasks. The novel motion tasks conceived in these studies are too ideal: they are always consistent patterns recorded while performing a particular gestural task, although all of the aforementioned studies claimed to increase MPR performance in practical application. However, in practice, complicated novel motion tasks are more likely to be involved in using an MPR system. When an intact-limbed user is equipped with a gestural interface using myoelectric control and common functional hand/finger tasks are selected as control commands (which is always the case), some daily activities, such as writing, typing, and mouse manipulation, produce novel patterns of other tasks occupying the same hand. They are similar to the target motion tasks, and therefore, it is difficult to identify and reject them by the control board. Meanwhile, prosthesis users may have the same problem when executing unconscious muscle contractions during daily movement. Thus, the aim of this study is to improve novelty rejection performance given complicated novel motion tasks in a real-life situation.

To alleviate the interference of unavoidable and complicated novel motion tasks, it is essential to implement sufficient information sensing for well-characterizing motion intentions and advanced methods for mining such information. In the field of sEMG recording and myoelectric control, the 2-dimensional flexible high-density electrode array has recently become popular. The recorded high-density sEMG (HD-sEMG) conveys additional spatial information rather than the routine temporal or spectral information inherent in the single-channel sEMG signal. Many studies achieved significant improvements in the myoelectric control performance using HD-sEMG (He and Zhu, 2017; Zhang et al., 2017; Cheng et al., 2018; Nougarou et al., 2019). However, how to mine spatial information is still an open question. Deep learning and a convolutional neural network (CNN) have recently revolutionized several fields of data science, such as natural language processing (Kim, 2014), computer vision (Krizhevsky et al., 2012), and reinforcement learning (Mnih et al., 2013). Also, many studies claimed a significant improvement in myoelectric control using CNN (Atzori et al., 2016; Wei et al., 2019). There is no doubt that CNN serves as a strong and useful tool for extracting spatial information to describe images. Thus, using CNN to describe HD-sEMG seems to be a reasonable and feasible choice. Furthermore, in the field of novelty detection, autoencoder has been recognized as a successful solution based on the assumption that it can model variable correlations of normal/target samples into a lower-dimensional subspace in which the abnormal/novel and target samples are significantly different (Sakurada and Yairi, 2014; Erfani et al., 2016; Zhou and Paffenroth, 2017). It is also straightforward to detect HD-sEMG pattern novelty using the autoencoder method.

In this study, the primary contribution is to resolve the interference from novel, changeable, and unpredictable motion tasks through applying hybrid neural networks to HD-sEMG data. The proposed framework of hybrid neural networks consists of two parts: one is a motion classification module using a CNN to characterize spatial information from HD-sEMG accurately, and the other is a novel motion detection module that employs autoencoder networks. Specifically, we first obtained feature representation of HD-sEMG using CNN and then learned inherent patterns of target samples with autoencoder networks. Thus, the sample from novel motion tasks can be identified by judging whether it can be fitted with any trained autoencoder.

## Methods

### Subjects

Nine volunteers were recruited to participate in the data collection experiment (six males, three females, all right-hand). The Ethics Review Board of the University of Science and Technology of China has approved the experimental protocol. The recruited subjects were intact-limbed and able-bodied without any known neuromuscular injury or disorder. They aged 25.11 ± 1.61 years [mean ± standard deviation (SD), ranged from 22 to 27 years]. The purpose and method of the experiment were orally described in detail to all individuals before any procedure of the experiment, and all subjects gave informed and signed consent.

### Experiments

**Figure 2:**
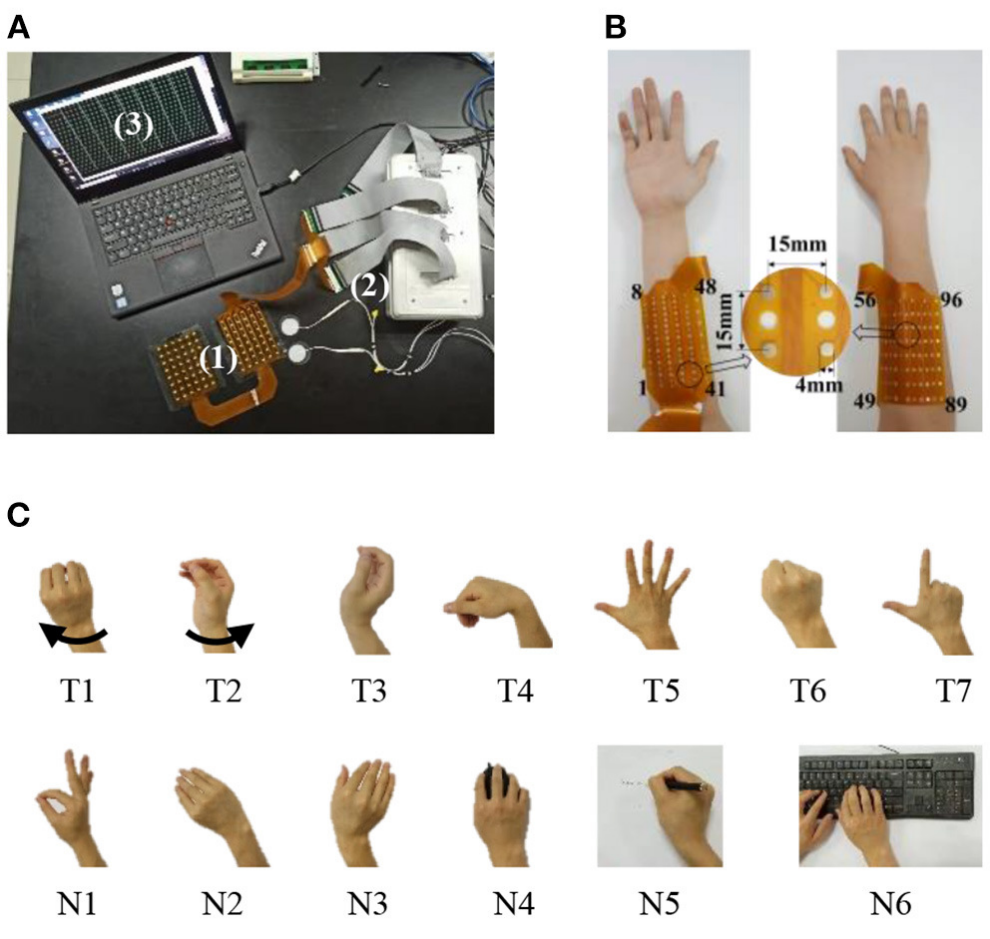
Experimental setup and scenario. **(A)** An overview of the experimental setup including electrode arrays concealed in the black armband (1), data recording device (2), and a computer with software interface to monitor the recorded signals in real time (3). **(B)** The placement of two pieces of high-density electrode arrays for collecting high-density surface electromyogram (HD-sEMG) data from forearm flexors and extensors. **(C)** An illustration of 13 motion tasks, including seven control/target motion tasks (T1–T7) and six interference/novel motion tasks (N1–N6).

As shown in Figure 2, two pieces of flexible high-density electrode array were developed to gather HD-sEMG signals from the forearm extensors and flexors. Each array has 48 monopolar sEMG channels that were organized in a 6 × 8 grid. The two arrays form a 12 × 8 matrix with 96 channels. The electrode diameter was 4 mm, and the interval between two consecutive electrodes was 15 mm. Also, a customized device was used to record and save sEMG signals, as shown in Figure 2A. The sEMG signals were initially amplified by a two-stage amplifier with a gain of 60 dB before being processed by a built-in band-pass filter (20–500 Hz). The signals were then sampled using a 16-bit analog-to-digital converter at a 1 kHz sampling rate. A USB cable was used to transport all digitalized data to a computer. During the experiments, the subjects sat comfortably in a height-adjustable chair. Before both electrode arrays were securely inserted, the subject's forearm skin was cleansed with 70% isopropyl alcohol. On both arms of the subject, two reference electrodes were attached to the olecranon.

We defined seven target motor tasks as the control commands of the MPR system, and they were wrist pronation (T1)/supination (T2), wrist extension (T3)/flexion (T4), hand opening (T5)/closing (T6), and shooting (T7). Then, six novel motion tasks were selected as control disturbances to the system. They were defined and grouped into two categories. One included simple and distinct motion tasks: pinch (N1), radial deviation (N2), and ulnar deviation (N3). Previous studies always considered motion tasks in this category. The other contained dynamic motion tasks: mouse manipulating (N4), handwriting (N5), and keyboard typing (N6). They were included as a result of common occurrences in regular office activity. All tasks are illustrated in Figure 2C. For each task of T1–T7 and N1–N3, each subject was required to perform isometric muscle contraction with 10 repetitions. For each repetition, subjects were asked to hold a mild muscular contraction for 5 s. For each task of N4–N5, subjects were instructed to do each activity for about 1 min to simulate a real-world scenario. In addition, enough rest between repetitions/tasks was given to minimize muscular fatigue.

### Novel Motion Rejection Using Hybrid Neural Network

**Figure 3:**
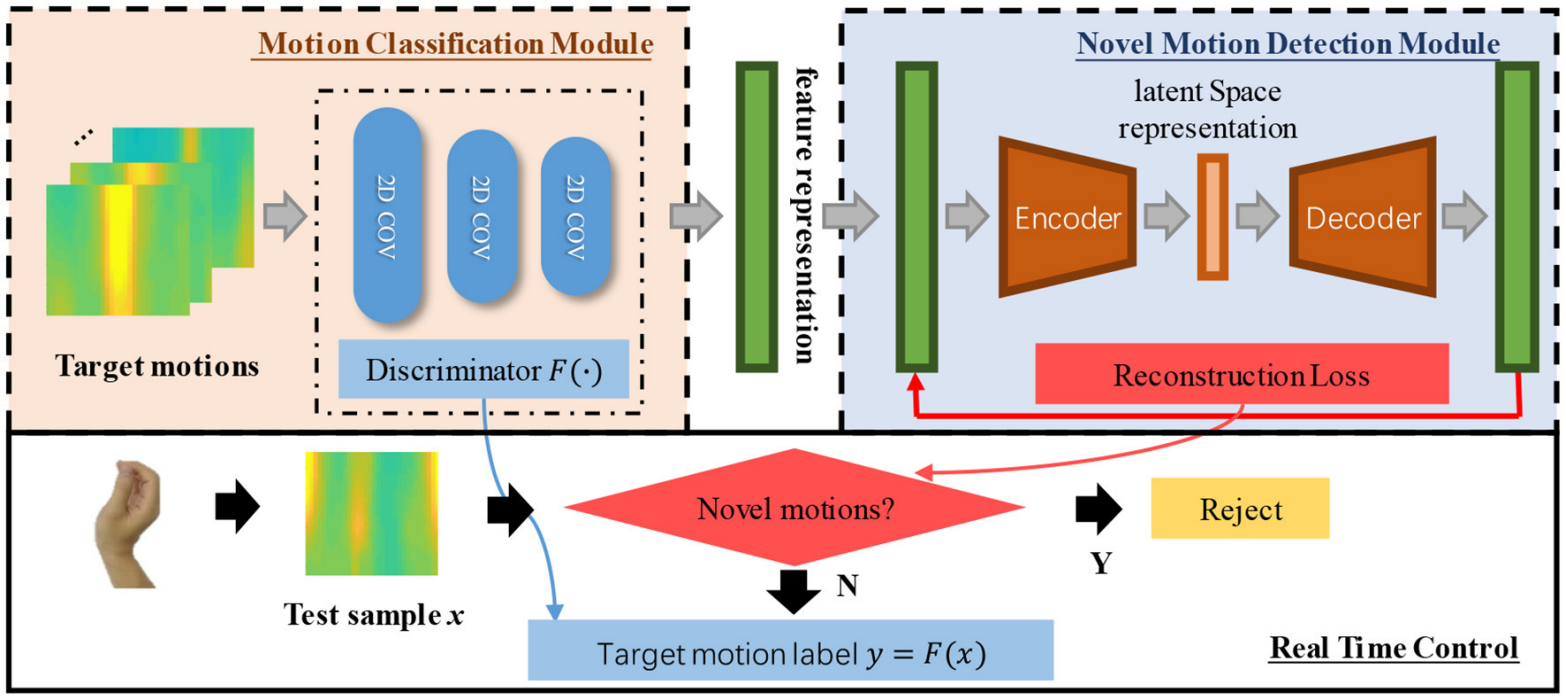
The proposed framework for target motion classification and novel motion rejection.

Figure 3 shows the architecture proposed in this study, which includes two modules, a motion classification module and a novelty detection module. The HD-sEMG signals are first processed into feature images, and the subsequent CNNs are adopted to identify the target motion tasks while further extracting feature representations based on inputted feature images. Next, the feature representations outputted by the CNNs are fed to an autoencoder network, which aims to reject any novel sample and, meanwhile, pass through target samples. Thus, if a test sample comes from one target motion task, the loss from the novel motion detection module should be small, and the final command is determined by the motion classification module. It is noted that only samples from target motion tasks are supplied to train the whole architecture. The details of the proposed method are described as follows.

#### Data Segmentation and Feature Extraction

The sEMG signals were first segmented into a series of overlapping windows, each having a window length of 250 ms and an increment of 150 ms. An amplitude thresholding approach was used to eliminate the windows from quiescent sEMG signals, with the threshold set to the mean plus three times the SD of the baseline signals averaged over all the channels. In this study, three typical time-domain (TD) features of the sEMG signals, namely mean absolute value (MAV), waveform length (WL), and root mean square (RMS), were chosen for their intuitive ability to depict muscle contraction intensity. Please note that both the MAV and the WL were selected from the well-known Hudgin's TD feature set. As a result, one window of the sEMG signal in the form of 12×8×250 was converted into a 12×8×3 feature matrix, with 12×8 representing channel distribution in the combination of both the 2-dimensional electrode arrays. Each processed feature matrix, equivalently viewed as a feature image, was considered as a basic sample in the following target pattern classification and the novel pattern detection analyses. At last, we randomly split the feature images (i.e., samples) of the target motion tasks into training, validation, and testing datasets with percentages of 64, 16, and 20%, respectively, to learn and fine-tune the model hyperparameters.

#### Motion Classification Module

**Figure 4:**
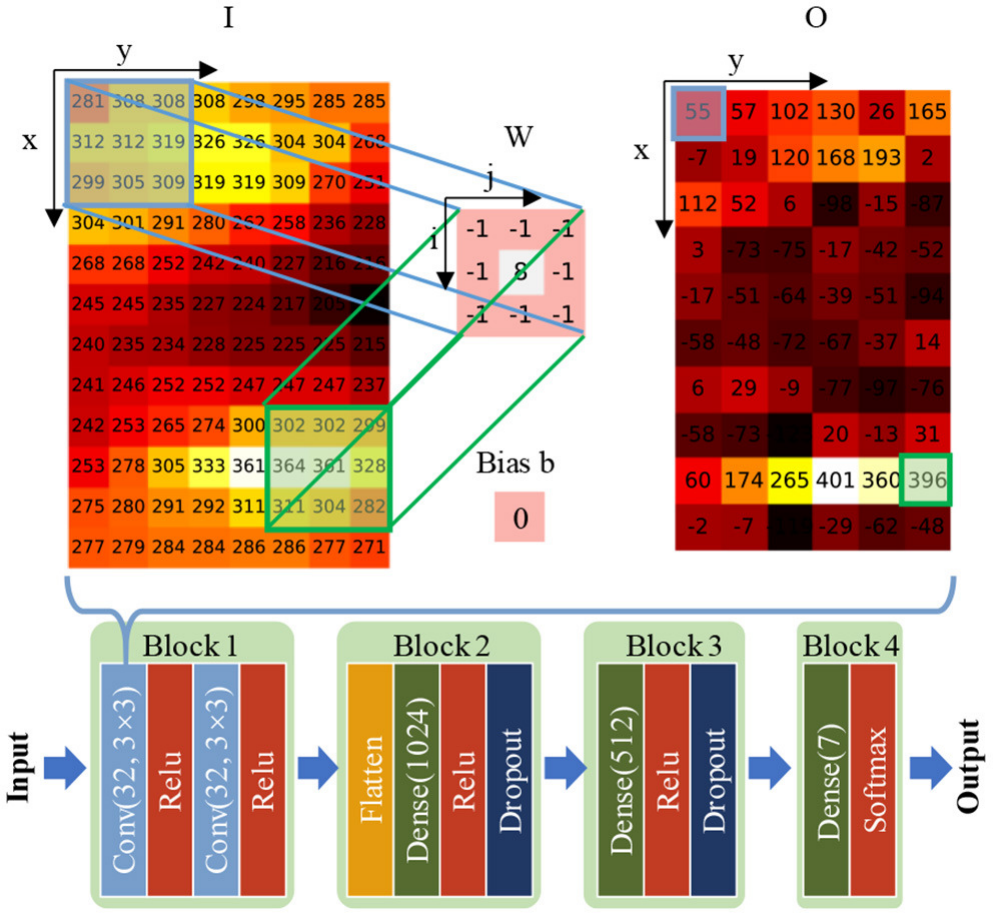
The configuration of the convolutional neural network (CNN) used in the motion classification module. Here, the “Conv” refers to a convolutional layer, and the two notations in the subsequent parentheses represent filter numbers and size in the current layer, respectively. One example of forward convolution pass is also illustrated in the upper part.

To better characterize the spatial information of HD-sEMG, the well-established CNN technology has been adopted in this module. The core advantage of the CNN is its ability to extract task-oriented spatial features autonomously and directly from inputted images, as shown in the upper part of Figure 4. The forward convolution pass is computed as


(1) $$O_{x,y}=f(\sum_{i}\sum_{j}\left(W_{i,j}\times I_{x+i,y+j})+b\right)$$

where *O*_*x,y*_ represents the value of output feature map at (*x, y*), *W*_*i,j*_ is the filter value at (*i,j*), *I*_*x* + *i, y* + *j*_ is the value at (*x*+*i, y*+*i*) of the inputted matrix, and b is the bias. In the upper part of Figure 4, the inputted I is the WL feature that comes from one sample of wrist pronation motion. Both forearm flexors and extensors produced strong muscular contraction, and the corresponding image exhibited two highlights in specific regions. Thus, the hypothetical sharpness filter W, which is likely to be learned by CNN, can effectively represent two strong activation zones and can restrain other flat areas, as shown in the matrix O. As a result, many adaptive spatial filters in the CNN can be combined to visualize image patterns such as sharpness, edges, and textures autonomously, which is hard to be characterized in a hand-craft manner.

The architecture of the neural network is demonstrated in the bottom part of Figure 4, consisting of four blocks. Block 1 of the net includes two convolutional layers and two ReLU (Krizhevsky et al., 2012) non-linear activation functions. Besides, 32 filters with 3 × 3 size are applied in each convolutional layer. In blocks 2–4 of the net, several dense layers with different numbers of nodes (the number in the subsequent parenthesis in Figure 4) are included. Activation functions of ReLU and Softmax are attached following the different dense layers. We also appended dropout layers with a 0.8 rate in blocks 2–3 to prevent overfitting (Srivastava et al., 2014).

Similar to the study by Tomczyński et al. (2015), the cross-entropy loss was adopted to help optimize the network parameters. The loss function is computed as


(2) $$\begin{array}{c} L=-\sum_{c=1}^{M}y_{x,c}\log\left(p_{x,c}\right) \end{array}$$

where M is the number of motion classes. *In the equation, y*_*x,c*_ represents a binary indicator (0 or 1), and it is equal to 1 only if the current sample x truly belongs to class c. The probability predicted by the net for the sample x belonging to class c is *p*_*x,c*_. The benefit of this function is to produce damped outputs for samples that are not related to specific motion tasks (Tomczyński et al., 2015). Next, we trained the network with mini-batch gradient descent using the AdaDelta algorithm (Zeiler, 2012). The batch size is set to 16, and the learning rate was 0.05 for 100 training epochs.

#### Novelty Detection Module

**Figure 5:**
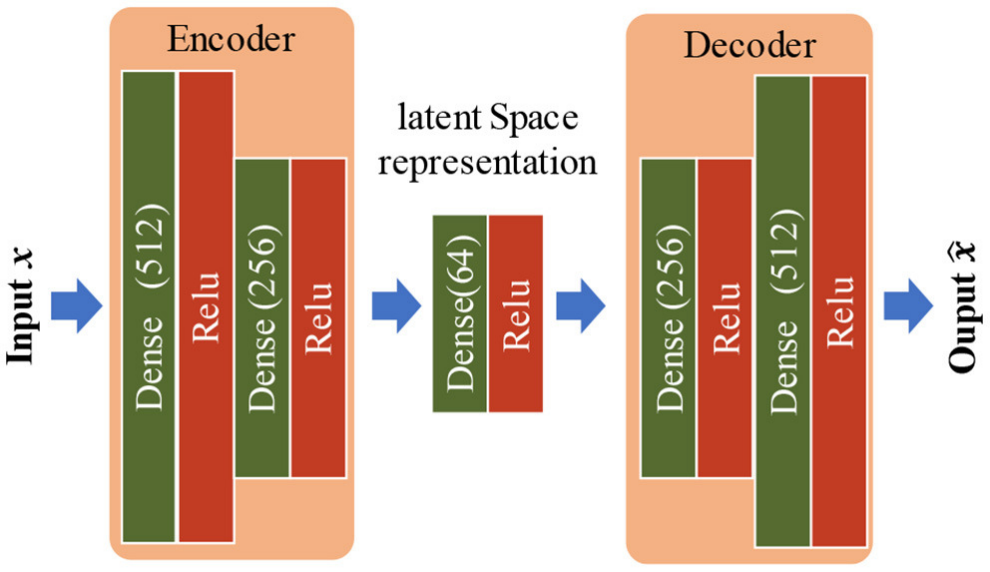
The configuration of the autoencoder adopted in the novelty detection module. Here, the number in the parentheses refers to the number of nodes of the layer.

Given the trained CNN from the previous module, the next step is to build autoencoder networks for detecting novel motion tasks. As shown in Figure 5, the autoencoder adopted in this study is constituted with several dense layers. There are two parts, both encoder and decoder, which commit to capturing inherent patterns of target motion tasks by embedding inputted data into a lower-dimensional subspace (Zhou and Paffenroth, 2017). For each target motion task, an autoencoder was built. The training set {**x**(1), **x**(2), …, **x**(*n*)} for one motion task, where **x**(*i*) ∈ ℝ^*D*^ represents a vector with D variables and n is the number of sample vectors, was assumed. This set was obtained as the outputs from block 3 of the well-trained CNN. These vectors were regarded as feature representations after inherent spatial information was well characterized, as mentioned in Figure 3. Thus, in this module, the input feature vector is in the form of 512×1 (the nodes of block 3 in the CNN is 512). In this study, the autoencoder only consisted of dense layers because it was one of the basic neural network components. The calculation between two consecutive dense layers is similar to the aforementioned forward convolution pass, and the details can be found in the study by Zhou and Paffenroth (2017).

During the training phase, we first compressed the training data into lower-dimensional latent space and subsequently reconstructed the output $\left\{\hat{\text{x}}\left(1\right),\hat{\text{x}}\left(2\right),\ldots ,\hat{\text{x}}\left(n\right)\right\}$. Empirically, the reconstruction error is computed *via* a mean squared error in the training phase, and it is assigned to be the Bray-Curtis distance (Bray and Curtis, 1957) in the testing phase, which is given as:


(3) $$E(i)=\frac{\sum_{k=1}^{D}\left|x_{ik}-{\hat{x}}_{ik}\right|}{\sum_{k=1}^{D}\left(x_{ik}+{\hat{x}}_{ik}\right)}$$

Many distance metrics are applicable to be used as the loss function. The above settings were determined through some pre-tests according to the optimal performance. We trained the network with mini-batch gradient descent using the stochastic gradient descent algorithm. The batch size was set to 16. The learning rate was initialized with 0.1 for the first 600 training epochs, and it decreased to 0.01 for another 300 epochs.

After the subspace was determined in the testing procedure, a testing sample was first projected into the subspace and was subsequently reproduced to reconstruct the original sample. The reconstruction error in Equation 3 is defined as an anomaly score, and the score is supposed to be low if the sample is truly from the target motion task, whereas the score is large with samples from novel motion tasks. Thus, for each target motion task, a threshold should be applied to the score to reject novel motion classes. In fact, there is a trade-off for the threshold determination. A lower threshold may reject more novel samples at the cost of unavoidable rejection of some target samples as well, while a higher threshold may weaken the power of novelty detection and rejection. In this study, we defined a recall factor describing the proportion of samples belonging to each target pattern that could be correctly identified as the labeled target pattern (which were not regarded to be the novelty). Evidently, the threshold of each target pattern was controlled by the recall factor: the threshold of each target pattern was set as the largest anomaly score among the correctly identified samples, given a specific recall factor; a higher recall factor led to a higher threshold for each task. In this study, we selected a consistent recall factor shared by all target motion tasks and subjects, and then thresholds for all tasks were determined accordingly. Given the validation dataset, a sensitivity analysis was conducted by adjusting the recall factor from 0 to 1. It was equivalent to adjusting the task-specific thresholds. The recall factor, as well as thresholds, could be finally set according to the optimal performance of the novelty rejection and task pattern classification.

After any testing sample was assigned to belong to any of the target motion tasks (other than novelty), its task pattern label was finally determined as the output of the motion classification module. The hybrid neural networks, i.e., the CNN and the autoencoder networks, were combined to solve the problem of novel motion rejection.

Furthermore, the architecture of all of the aforementioned networks was defined by a series of pre-tests to get optimal results. All of the algorithms were written in Python using the Keras framework, and they were tested on a laptop with an Intel Core i7 processor, 16 GB of RAM, and an NVIDIA GeForce GTX 1050 Ti GPU. The source codes are published online for further clarification.

### Performance Evaluation and Statistical Analysis

To get an intuition of how the features extracted from CNN are arranged in a high-dimensional space, t-distributed stochastic neighbor embedding (t-SNE) (van der Maaten and Hinton, 2008) was applied for visualization. The t-SNE is a powerful algorithm for dimensionality reduction that is well-suited for embedding high-dimensional data for visualization in the space of two or three dimensions. The algorithm calculates a similarity measure between pairs of samples in the high and low-dimensional spaces. As a result, comparable samples are modeled by neighboring points with a high probability, whereas dissimilar samples are modeled by distant points.

Further, two common MPR methods were implemented for performance comparison against the proposed method. One was a conventional MPR method using a TD feature set and an LDA classifier (Englehart and Hudgins, 2003), denoted as the LDA method in this study. The other method was suggested in Amsuess et al. (2015) and Ding et al. (2017), which combined the LDA method with MD to expand its capability of novelty rejection. It was denoted as the LDA-MD method, and it was used to transform the feature data using LDA before calculating the MD. Similar to the proposed method in this study, the MD value was regarded as an anomaly score reflecting the probability of the sample belonging to target motion tasks. On this basis, a threshold needed to be applied. Therefore, we used the same recall factor to control the setting of the threshold, given the validation dataset. When implementing both comparison methods, four TD features (Englehart and Hudgins, 2003) were extracted from each sEMG channel. Then, for each window, all features were concatenated into a 384-dimensional (96 × 4) vector.

To evaluate the novelty rejection performance and to establish criteria for setting appropriate thresholds (involved in both the LDA-MD method and the proposed method), a receiver operating characteristic (ROC) curve was obtained. Plotting the true positive rate (TPR) against the false positive rate (FPR) at various threshold levels yields the ROC curve. This curve gives a plot of the threshold effect on the accuracies for identifying target/novel samples. The ROC curve's area under the curve (AUC) was utilized as a metric, which indicates how well a model can differentiate between classes. A higher AUC represents the better performance of correctly distinguishing novelty from all target motion tasks. In addition, after the threshold was determined according to the ROC curve, the classification accuracy of each motion can be calculated as


(4) $$\begin{array}{c} Acc=\;\frac{Number\;of\;correctly\;classified\;samples}{Number\;of\;all\;samples} \end{array}$$

where samples of six novel motion tasks shared just one label, i.e., the novelty.

Multiple paired samples *t*-test was applied on AUC or accuracy to compare novelty rejection performance of both the LDA-MD method and the proposed method, respectively. In addition, a one-way repeated-measure ANOVA was applied on accuracies to examine the effect of the method (three observation levels: the LDA method, the LDA-MD method, and the proposed method) on motion classification performance. *Post-hoc* multiple comparisons with LSD adjustments were made if necessary. The significance level for this study was set at 0.05. SPSS software was used for all statistical analyses (ver. 24.0, SPSS Inc. Chicago, IL, USA).

## Results

### Feature Visualization With t-SNE

**Figure 6:**
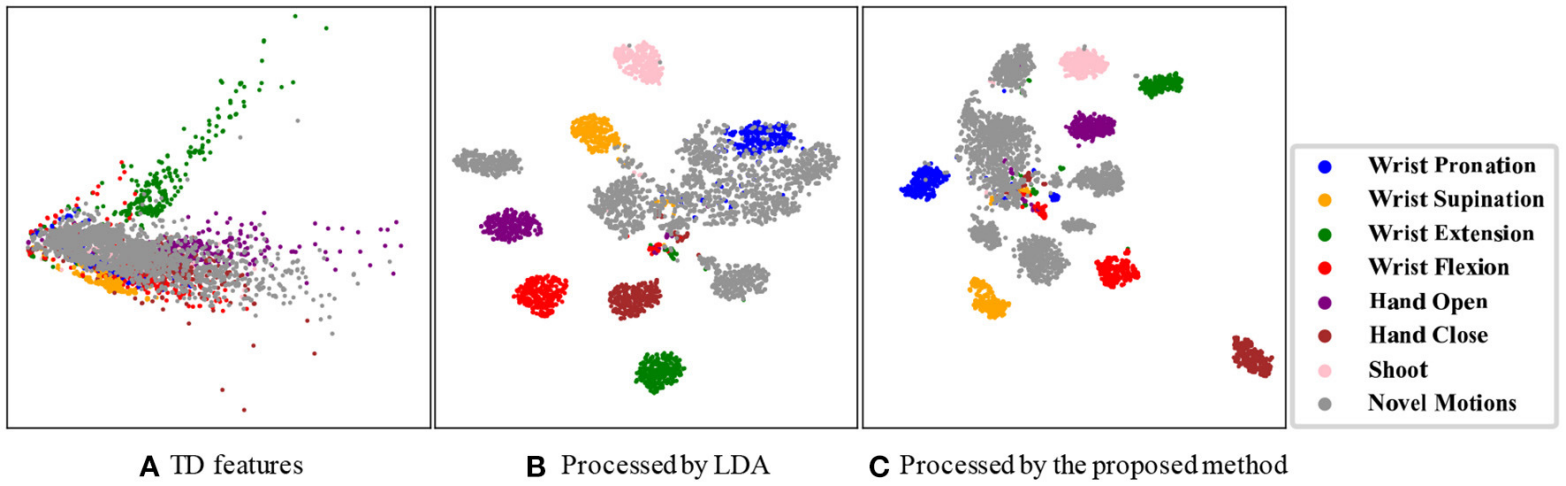
Distribution of different feature sets visualized by a t-distributed stochastic neighbor embedding (t-SNE) algorithm. All data samples from one representative subject are used as an example.

Figure 6 provides an insight about the class separability when the data are characterized by the TD feature set, the TD feature set processed by the LDA, and the feature representation obtained by the CNN in the proposed method. We can observe that the TD feature set exhibited weak separability between the target and novel samples, with almost all classes overlapping with each other seriously. When the LDA method further processes the TD features, it was found that samples from each of the target motion tasks were concentrated into a small region, while the samples from novel motion tasks were scattered around. However, the partial samples from wrist pronation and novel motion tasks were still mixed. Finally, when applying the proposed method, we noticed that the samples were well separated by their true target motion labels and that samples from novel motion tasks were scattered around.

### Performance of Rejecting Novel Motion Tasks

**Figure 7:**
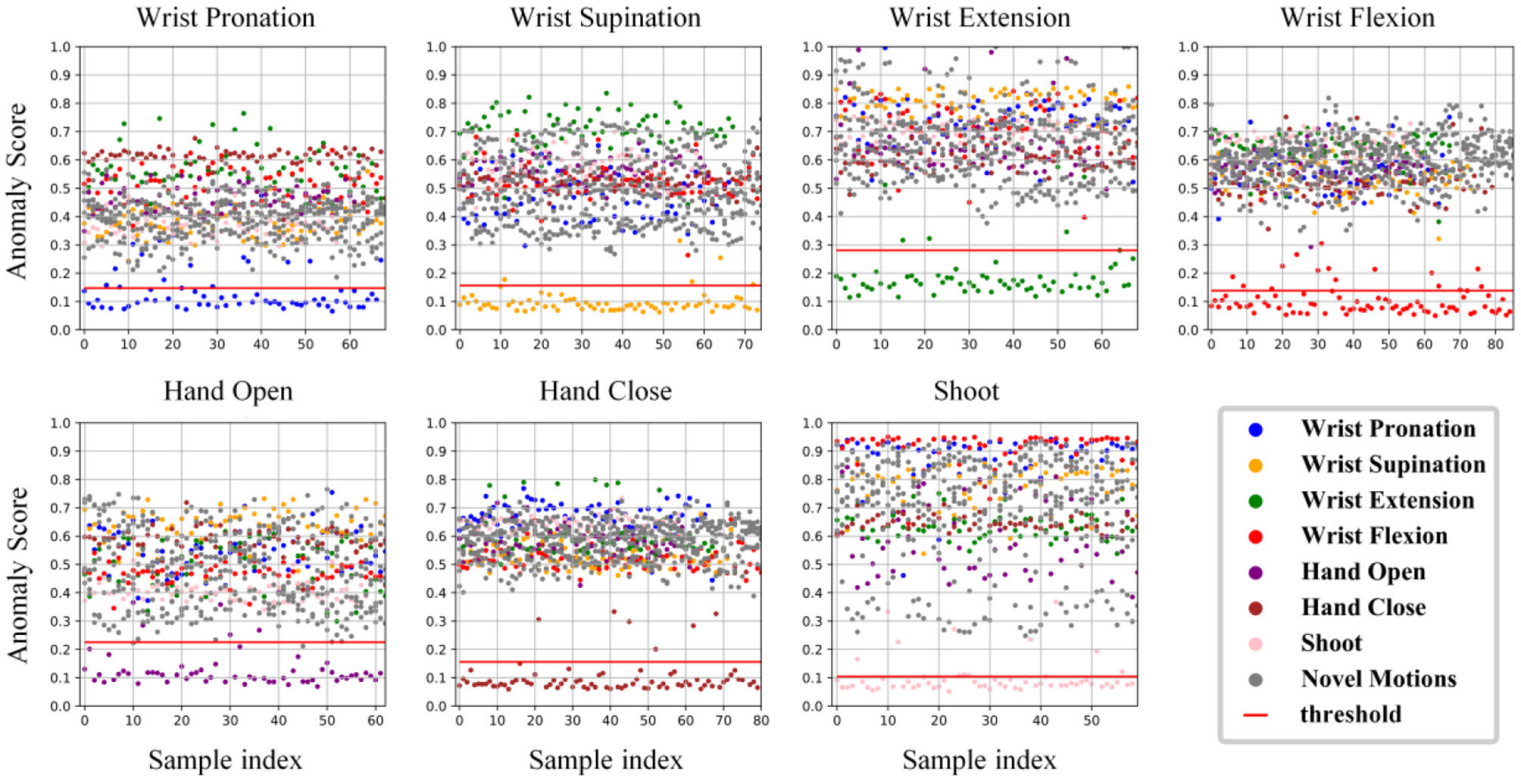
Anomaly scores, i.e., reconstruction errors, derived from applying seven autoencoders on the testing dataset from one representative subject, respectively. Each autoencoder was only trained with the data from one target motion task. The threshold for each autoencoder is shown in a red horizontal line. Please note that the threshold was determined from the validation dataset using samples of the corresponding task, while all samples in the testing dataset (including both target and novel tasks) are shown in each subplot, to demonstrate the usability of our strategy for determining thresholds.

As shown in Figure 7, when samples of one target motion task were fed into their corresponding well-trained autoencoder, the anomaly scores, i.e., reconstruction errors from Equation 3, were quite minor, whereas samples from other motion tasks (including other remaining target motion tasks and the novel motion tasks) had much bigger scores. Thus, for each auto-encoder, an appropriate threshold was required to identify most of the corresponding target samples and meanwhile to reject other samples.

**Figure 8:**
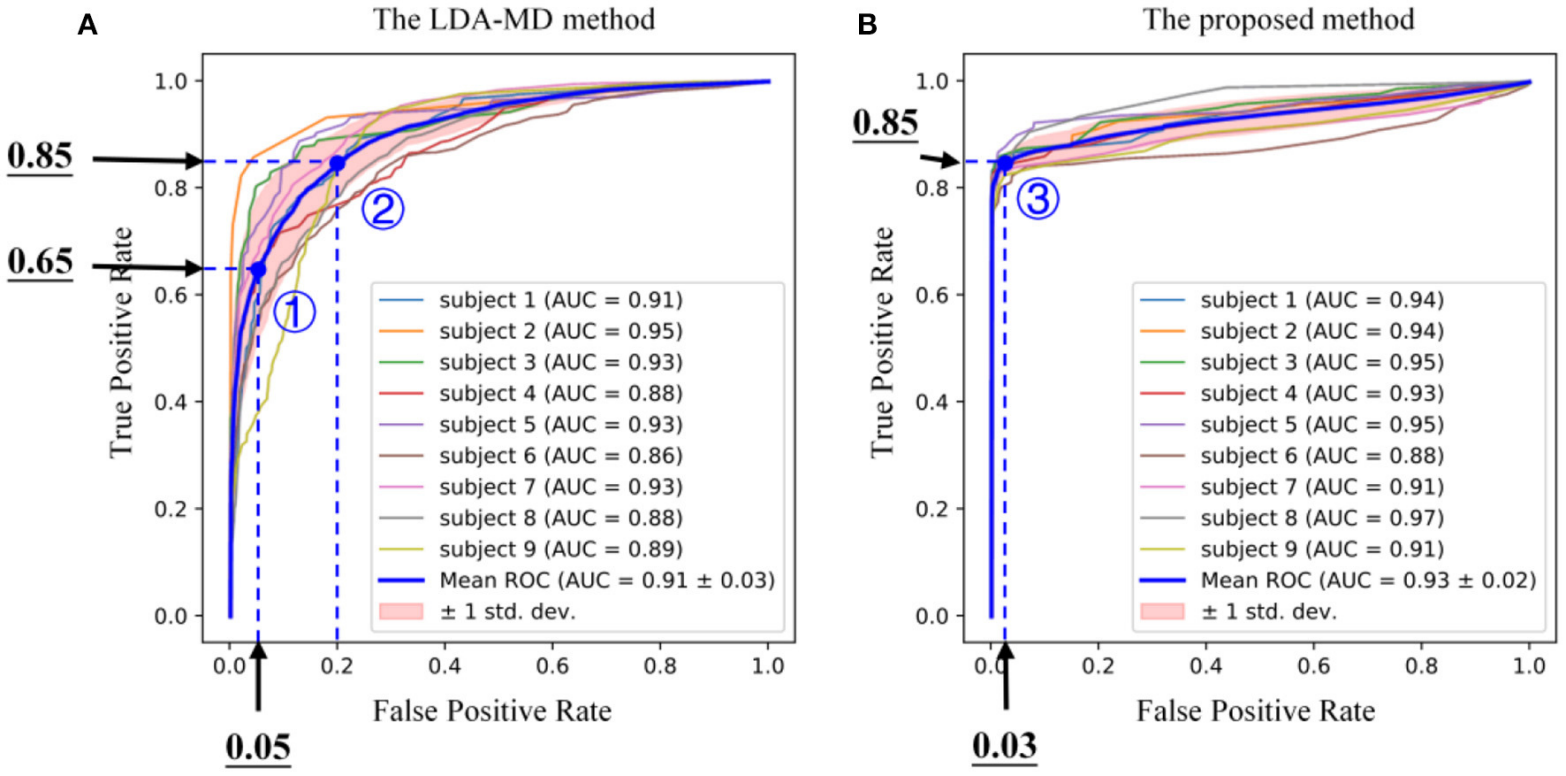
The received operating curves (ROC) derived from the testing datasets for different subjects using the routine Fisher linear discriminant analysis and the Mahalanobis distance (LDA-MD) method **(A)** and the proposed method **(B)**, respectively. In each subplot, the bold blue curve is the mean curve averaged across all subjects and the pink region represents the standard deviation. Three points in the curves marked with circle numbers indicate different threshold settings. The LDA-MD method yielded a low true positive rate (TPR) of 0.65 at a low false positive rate (FPR) of 0.05 (the point 1), and its TPR was improved to 0.85 at a FPR of 0.2 (the point 2). By contrast, the proposed method had a TPR of 0.85 at a very low FPR of 0.03 (the point 3).

Figure 8 illustrates the ROC curves derived from the testing dataset for different subjects when both the LDA-MD method and the proposed method were applied (we omitted the LDA method because of its lack of ability in rejecting novel motion tasks). The ROC curve showed the trade-off between sensitivity (i.e., TPR) and specificity (i.e., 1-FPR) under different threshold settings. The AUC values obtained by the proposed method were significantly higher than those obtained by the conventional LDA-MD method (0.91±0.03 for the LDA-MD method vs. 0.93±0.02 for the proposed method, *p* < 0.05). Besides, the “steepness” of ROC curves was also critical since it was ideal for maximizing the TPR while minimizing the FPR. It was found that the mean ROC curve of the proposed method was able to approximate the top-left corner (high TPR under the condition of low FPR) as compared with that of the LDA-MD method, indicating a performance improvement. In practice, appropriate thresholds need to be set to keep a low FPR of no more than 5% to ensure a high level (about 95%) of novelty rejection accuracy. According to Figure 8B, the proposed method was able to yield a low FPR of around 3%, whereas the TPR was about 85%. Considering that the recall is the TPR by definition, we intentionally set the recall factor at 0.85 for the appropriate setting of thresholds. Please note that both the recall factor and the TPR were calculated with the validation and testing datasets, respectively. Therefore, they were slightly different in values due to the same distribution of target samples in both datasets. In the following analyses, we set thresholds for all target motion tasks by means of applying a consistent recall factor of 0.85 to the validation dataset when both methods were used.

### Performance of Identifying All Motion Tasks

Table 1 illustrates the average classification accuracies of all the subjects using three methods. When compared to the routine LDA method, the LDA-MD method and the proposed method achieved lower classification accuracies of target motion tasks (95.30±2.00% for the LDA method, 84.13±2.22% for the LDA-MD method, and 83.00±1.60% for the proposed method, *p* < 0.05 for comparisons between any two of the three methods). There were no differences between the LDA-MD and the proposed method according to the one-way repeated-measure ANOVA (*p* = 0.87). In terms of the rejection of novel motion tasks, however, the routine LDA method failed to reject any novel sample, with an accuracy of 0%. By contrast, the LDA-MD and the proposed methods were able to identify and subsequently reject novel motion tasks (63.00±29.44% vs. 97.33±3.67% for the pinch motion, 95.22±3.51% vs. 98.77±1.64% for the radial deviation motion, 90.00±11.12% vs. 95.55±7.74% for the ulnar deviation motion, 80.96±19.63% vs. 99.44±1.13% for the mouse manipulation, 78.77±17.00% vs. 97.22±7.59% for writing, and 75.25±20.79% vs. 98.44±1.94% for typing). Also, the paired samples t-test informed the proposed method significantly outperformed the LDA-MD method (*p* < 0.05 for all comparisons).

**Table 1:** Classification accuracies (%) of identifying the target and novel motion tasks averaged across all subjects using three methods, respectively.

**Method**	**Target motions**	**Novel motions**					
	**Avg**.	**Pinch**	**Radial deviation**	**Ulnar deviation**	**Mouse manipulating**	**Writing**	**Typing**
LDA	**95.30**	0	0	0	0	0	0
LDA-MD	84.13	63.00	95.22	90.00	80.96	78.77	75.25
The proposed method	83.00	**97.33**	**98.77**	**95.55**	**99.44**	**97.22**	**98.44**

*The bold values indicate the highest value for each column*.

**Figure 9:**
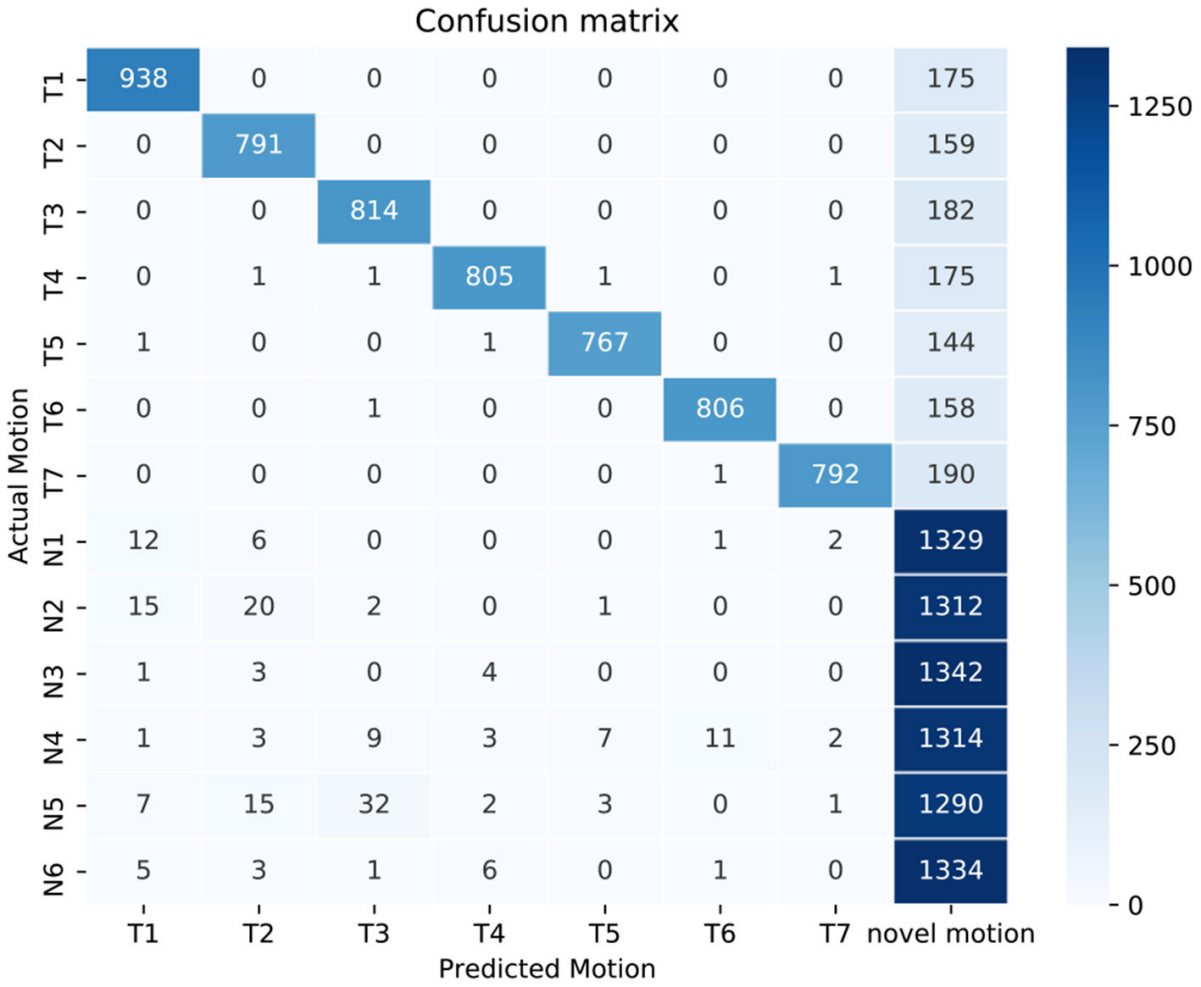
A confusion matrix in the prediction of all motion tasks using the proposed method, when the recall factor is set to 0.85. The data samples from all subjects are summarized in the same matrix. T1–T7 indicate seven target motion tasks, and N1–N6 indicate six novel motion tasks.

The confusion matrix is shown in Figure 9 in the prediction of target motion tasks (T1–T7) and novel motion tasks (N1–N6). The proposed method can accurately detect novel motion tasks (> 95% for all novel motion tasks). Besides, we surprisingly found that, although some samples from target motion tasks were misclassified, most of these samples were predicted as novel motion tasks, other than misclassifications to other target motion tasks.

**Figure 10:**
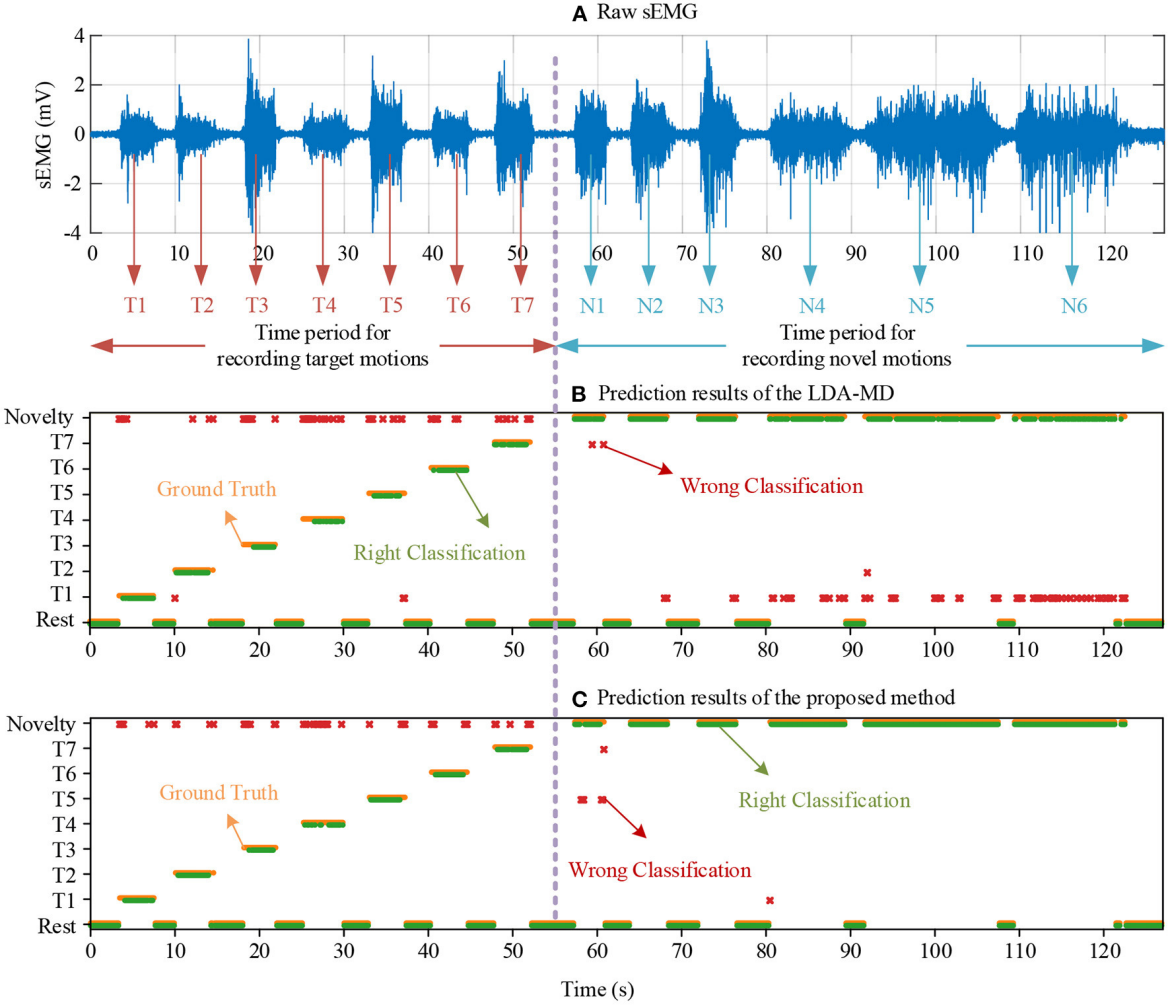
An example of the raw one-channel sEMG signal from a representative subject **(A)** and its corresponding classification decisions using the LDA-MD method **(B)** and the proposed method **(C)**, respectively. T1–T7 indicate seven target motion tasks, and N1–N6 indicate six novel motion tasks. The orange dots are the “ground truth” of task patterns; the green dots represent correct classifications; and the red asterisks indicate some wrong classifications.

Figure 10 depicts a representative example of a raw sEMG signal in one channel, as well as recognition decisions from the LDA and the proposed method. The LDA-MD method failed to reject these novel motion tasks in time during the complicated motion tasks, such as mouse manipulation, writing, and typing. On the other hand, the proposed method can detect and reject samples from novel motion tasks with pinpoint accuracy. Also, we found that the misclassifications using the proposed method were prone to occur at the transient phase of different target motion tasks.

## Discussion

Novel motion interference is a major issue that limits the practical applications of myoelectric control systems in the creation of human–machine interfaces. In this study, hybrid networks that consist of CNN and autoencoder networks are proposed to alleviate the interference. First, we utilize CNN to extract spatial information from HD-sEMG. Recently, the emergence of the HD-sEMG techniques has injected new vitality into the field of myoelectric pattern-recognition control. The extra spatial information derived from HD-sEMG offers more benefits for overcoming specific bottleneck problems in the myoelectric control than the mainly temporal information conveyed by the sEMG time series collected from typical separate electrodes. Figure 6 demonstrates the separability of the CNN feature representations, where most samples of target motion tasks are clustered into some small regions, and samples of novel motion tasks are very likely to be scattered into the blank area between those regions, facilitating the discrimination between the target motion patterns and the novelty patterns. This phenomenon proves our hypothesis that the use of CNN as a powerful tool for characterizing spatial information in feature images redounds to identifying different patterns, no matter whether they are learned or not.

Given the feature representations from CNN, the next challenge is to reject novel motion samples. It is difficult to find statistical correlations from the data with high dimensions. In this study, thus, autoencoder is applied because of its advantage in non-linear novelty detection of high dimensional data (Sakurada and Yairi, 2014). Figure 7 shows that all autoencoders can capture the correlation of the inherent variables within their training data, and only the testing data from the given motion can be fitted with the trained model. However, given each well-trained autoencoder, some samples truly from the target motion task still had exceptionally higher anomaly scores. One possible explanation is a data variation, such as instantaneous variations in the non-stationary physiological signal (i.e., sEMG) and unconscious changes during voluntary muscle contractions (e.g., force level fluctuations), although all subjects were instructed to hold each target motion task at a stable and a medium force levels. Such data variations are usually unavoidable, especially at the transient phases of the task motion performance. Given the data variations, a portion of samples may be regarded as outliers. Nevertheless, due to the good performance of the autoencoder, the anomaly scores from the training motion task and other motion tasks could be well separated only by a simple threshold, where most samples from the training motion task can be reserved while the samples from the rest motion tasks can be completely discarded. From the ROC curves reported in Figure 8, the proposed method was found to outperform the routine LDA-MD method, with the AUC improved significantly from 0.91 to 0.93 (*p* < 0.05). Further, from the “steepness” of ROC curves, we found that the most meaningful contribution of the proposed method was its ability to maintain a high TPR (around 85%) while achieving a very low FPR (close to 0%). This implies that there exists a recall factor as well as its determined thresholds, which can identify and reject almost all novel motion tasks completely at a very low cost of losing a small portion of target samples. Nevertheless, although the routine LDA-MD method was able to achieve a relatively high AUC (above 90%), approximating to that of the proposed method, it failed to keep a low FPR while maintaining a high TPR. Its inferior performance in novelty rejection may be attributed to the limited capability of well characterizing spatial information in feature images. The LDA-MD method flattened each feature image into a vector, probably leading to the loss of useful spatial information. In addition, the MD approach employed a simple rejection strategy, and it was not able to mine variable correlations adequately.

According to the classification accuracies of different methods, although the routine LDA method achieved high performance with around 95% accuracy when identifying target motion tasks, it cannot reject any novel motion tasks. With the ability of novelty rejection, the LDA-MD (Amsuess et al., 2015; Ding et al., 2017) and the proposed method have to sacrifice a portion of target samples, which may have ambiguous patterns and be inconsistent with most samples in the training dataset. These ambiguous target samples are very likely to be correctly identified by the LDA method that divides the whole feature space into multiple subspaces without considering the problem of novelty interference and predicts all samples as target patterns according to the class probabilities. Thus, compared to the LDA method, the LDA-MD method achieved a little bit lower accuracy for the classification of target motion tasks (around 85%), and this is also the case for the proposed method, without any difference between them (*p* = 0.87). However, in terms of rejecting novel motion tasks, the LDA method inevitably misclassifies all novel samples into target motion tasks. In addition, the proposed method obtained high accuracies of around 97% across all tasks, and it outperforms the routine LDA-MD method with statistical significance (*p* < 0.05).

Besides, the relatively low classification accuracies of target motion tasks obtained by the proposed method can also be attributed to the strict threshold adopted in this study. This threshold was chosen intentionally to suppress the FPR approximating to 0%, resulting in an unavoidably decreased tolerance to data variations of the target samples. The confusion matrix in Figure 9 also supports the previous assumption that almost all the misclassified samples of target motion tasks are predicted to be the novel motion. Further, it was also reasonable to find that these misclassified target samples mainly occurred in the task transient-phase (Figure 10) because the motion task pattern in these periods tended to change and became ambiguous. In practice, the MPR control system can make a series of decisions in an interval of very short time (in a window length of 250 ms) at a relatively high frequency (at a frequency of 6.67 Hz given a window increment of 150 ms) to ensure continuous control. When performing a specific target motion pattern, the occasional misclassifications into the novelty are ephemeral, and they are rejected by the controller without any response. Therefore, such occasional commands missing in a stream of correct commands may not affect the user's experience, which may be easily compensated with appropriate post-processing methods designed for correcting and smoothing the control commands.

It is worth noting that the novel motion tasks simulated in this study are much more complicated compared to previous studies (Scheme et al., 2011; Amsuess et al., 2015; Tomczyński et al., 2015; Ding et al., 2017, 2019). It can be found that most previous studies mainly simplified the novelty detection issue: each novel motion task was performed with an isometric muscle contraction producing a consistent pattern. In this study, six novel motion tasks were investigated, including three “static” motion tasks with isometric muscle contractions (they were consistent with those in previous studies) and additional three dynamic motion tasks. Given this database, the conventional LDA-MD method yielded satisfactorily high accuracies for rejecting both static and stable novelty patterns, namely the ulnar deviation and the radial deviation (95.22±3.51% and 90.00±11.12%, respectively). This finding in the current study was consistent with previous reports (Amsuess et al., 2015; Ding et al., 2017). When the number of novel motions increased, however, the LDA-MD method failed to correctly reject the pinch task with a low accuracy of 63.00±29.44% due to its similarity in pattern with other target motion tasks. Furthermore, many samples from three dynamic motion tasks were prone to be misclassified by the LDA-MD method into the wrist pronation task, which led to low accuracies (from 75.35 to 80.96%). The reason may be attributed to the simultaneous involvement of the wrist pronation pattern in the dynamic novel motion tasks (i.e., mouse manipulating, writing, and typing), which were believed to involve larger pattern variations. By contrast, the proposed method outperformed the LDA-MD method by improving the accuracies of rejecting all novel motion tasks to a much higher level of around 95%. Given the challenging protocol with enormous data variations in dynamic novelty patterns, the proposed method still achieved satisfactory performance, further demonstrating its advance in overcoming a variety of novel interferences in real applications.

Overall, the attributes of the proposed method are summarized in Table 2 to emphasize its uniqueness compared to prior methods. It should be noted that the proposed method can effectively identify and target novel motion tasks due to its good ability to mine spatial information.

**Table 2:** The comparison of the proposed method and previous works.

**Method category**	**Representatives**	**Capabilities**		
		**Static novel motion tasks**	**Dynamic novel motion tasks**	**Mining spatial information**
Confidence based	• Scheme et al., 2013	×	√	×
	• Tomczyński et al., 2015	√	×	×
	• Robertson et al., 2019	×	√	×
One-vs.-all classification rule	• Liu and Huang, 2009	√	×	×
	• Scheme et al., 2011	√	×	×
	• Amsuess et al., 2015	√	×	×
	• Ding et al., 2017	√	×	×
	• Ding et al., 2019	√	×	×
	•**The proposed method**	**√**	**√**	**√**

*“×” means not to support the capability; “√” means to support the capability*.

Finally, there are several limitations in the present work. First, some misclassifications in transient and sustained phases of myoelectric signals do not make the control process smooth. Thus, some advanced technologies, such as the classification of transient signals (Kanitz et al., 2018) and advanced post-processing approaches (Yu et al., 2019), may help to improve the user experience. Besides, we regard each data sample at each time index to be independent, i.e., we might ignore temporal correlations within each myoelectric pattern. Although the performance is already sufficient without such sequential or temporal information, we can try some video processing methods by considering temporally correlated information (Simonyan and Zisserman, 2014). In addition, the proposed method achieves inferior accuracy in classifying target motions compared with the LDA method. The ideal solution should achieve high classification accuracy for both target and novel motions. The ongoing study is performed to construct more robust methods to improve the rejection performance while improving the ability to classify target motions. At last, the field of robot control is gradually attracting attention (Luo et al., 2019, 2021; Su et al., 2020a,b). It is no doubt that rejecting novel motions while controlling robots will increase the robustness. In future, we will evaluate the proposed method under real robot control scenarios. These topics will be the focus of our future work.

## Conclusion

This study proposed a method for alleviating novel motion interference toward achieving robust myoelectric pattern-recognition control. A framework using hybrid neural networks, i.e., CNN and autoencoder networks, were adopted. The CNN was used to characterize spatial information from HD-sEMG. Then, the autoencoder networks are applied to learn variable correlations in the spatial information and subsequently to reject novel motion tasks. The proposed method achieved high rejection accuracies while the routine methods failed to correctly reject novel motion tasks, demonstrating that it is a promising solution for novel motion rejection that can be used to enhance the robustness of myoelectric control systems.

## Data Availability Statement

The original contributions presented in the study are included in the article/Supplementary Material, further inquiries can be directed to the corresponding author/s.

## Ethics Statement

The studies involving human participants were reviewed and approved by the Ethics Review Board of University of Science and Technology of China. The patients/participants provided their written informed consent to participate in this study.

## Author Contributions

LW: methodology, software, validation, analysis, and writing-original draft. XuC: conceptualization and investigation. XiC: visualization. XZ: conceptualization, writing-review and editing, and supervision. All authors contributed to the article and approved the submitted version.

## Funding

This work was supported in part by the National Natural Science Foundation of China under Grants 61771444 and 61922075.

## Conflict of Interest

The authors declare that the research was conducted in the absence of any commercial or financial relationships that could be construed as a potential conflict of interest.

## Publisher's Note

All claims expressed in this article are solely those of the authors and do not necessarily represent those of their affiliated organizations, or those of the publisher, the editors and the reviewers. Any product that may be evaluated in this article, or claim that may be made by its manufacturer, is not guaranteed or endorsed by the publisher.

## Supplementary Material

Supplementary Video 1 Demo Video.
